# Targeting the centriolar replication factor STIL synergizes with DNA damaging agents for treatment of ovarian cancer

**DOI:** 10.18632/oncotarget.16068

**Published:** 2017-03-10

**Authors:** Noa Rabinowicz, Lingegowda S. Mangala, Kevin R. Brown, Cintia Checa-Rodriguez, Asher Castiel, Oren Moskovich, Giulia Zarfati, Luba Trakhtenbrot, Adva Levy-Barda, Dahai Jiang, Cristian Rodriguez-Aguayo, Sunila Pradeep, Yael van Praag, Gabriel Lopez-Berestein, Ahuvit David, Ilya Novikov, Pablo Huertas, Robert Rottapel, Anil K. Sood, Shai Izraeli

**Affiliations:** ^1^ Cancer Research Center, Sheba Medical Center, Tel Hashomer, Ramat Gan, Israel; ^2^ Department of Human Molecular Genetics and Biochemistry, Sackler Faculty of Medicine, Tel Aviv University, Tel Aviv, Israel; ^3^ Department of Gynecologic Oncology, MD Anderson Cancer Center, Houston, Texas, USA; ^4^ Center for RNA Interference and Non-Coding RNA, MD Anderson Cancer Center, Houston, Texas, USA; ^5^ Donnelly Centre and The Banting and Best Department of Medical Research, University of Toronto, Toronto, Ontario, Canada; ^6^ Department of Genetics, University of Sevilla and Centro Andaluz de Biología Molecular y Medicina Regenerativa (CABIMER), Sevilla, Spain; ^7^ Department of Experimental Therapeutics, MD Anderson Cancer Center, Houston, Texas, USA; ^8^ Department of Cancer Biology, MD Anderson Cancer Center, Houston, Texas, USA; ^9^ Biostatistical Unit, Gertner Institute for Epidemiology and Health Policy Research, Ramat Gan, Israel; ^10^ Princess Margaret Cancer Center, University Health Network, Toronto, Ontario, Canada; ^11^ The Gene Development and Environment Pediatric Research Institute, Edmond and Lily Safra Children's Hospital, Sheba Medical Center, Tel Hashomer, Ramat Gan, Israel

**Keywords:** STIL, centrosomes, ovarian cancer, DNA damage, genomic instability

## Abstract

Advanced ovarian cancer is an incurable disease. Thus, novel therapies are required. We wished to identify new therapeutic targets for ovarian cancer. ShRNA screen performed in 42 ovarian cancer cell lines identified the centriolar replication factor STIL as an essential gene for ovarian cancer cells. This was verified *in-vivo* in orthotopic human ovarian cancer mouse models. STIL depletion by administration of siRNA in neutral liposomes resulted in robust anti-tumor effect that was further enhanced in combination with cisplatin. Consistent with this finding, STIL depletion enhanced the extent of DNA double strand breaks caused by DNA damaging agents. This was associated with centrosomal depletion, ongoing genomic instability and enhanced formation of micronuclei. Interestingly, the ongoing DNA damage was not associated with reduced DNA repair. Indeed, we observed that depletion of STIL enhanced canonical homologous recombination repair and increased BRCA1 and RAD51 foci in response to DNA double strand breaks. Thus, inhibition of STIL significantly enhances the efficacy of DNA damaging chemotherapeutic drugs in treatment of ovarian cancer.

## INTRODUCTION

The SCL TAL1 Interrupting Locus (STIL, previously named SIL) protein is essential for the replication of centrioles, which are the core structures of centrosomes. Centrioles are important for chromosome segregation during mitosis and for the structure and function of primary cilia [1–4]. STIL is a 150KD protein conserved in vertebrates that is thought to act as a scaffold to many centrosomal, mitotic and cilia related proteins [5, 6, 7, 8]. STIL is expressed only in proliferating cells and its expression in cancer cells is associated with metastasis and worse prognosis. [9–13].

Ovarian cancers are often diagnosed at advanced stages with intraperitoneal spread. Although transient remissions may be achieved with chemotherapy, the prognosis of advanced ovarian cancer is grim [14]. Thus, newer therapies are needed. Centrosomes are often abnormal in cancer, including ovarian cancer [15–20] and centriolar amplification was recently shown to promote carcinogenesis [21]. Hence, centrosome-regulating proteins have been proposed as targets for therapy [17, 22–27]. Here we report that an unbiased shRNA screen identified STIL as essential for ovarian cancer cell survival. Using *in-vitro* and *in-vivo* orthotopic ovarian cancer pre-clinical models, we validated these findings and further show that STIL enhances the anti-tumor efficacy of DNA damaging chemotherapy. Thus, suppression of STIL may augment the effectiveness of current chemotherapeutic drugs used to treat this deadly malignancy.

## RESULTS

### STIL as a target for therapy of ovarian cancer

**Figure 1:**
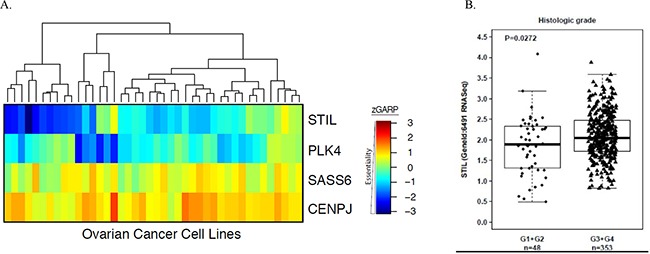
STIL is highly expressed in ovarian cancer tumors and is essential for the survival of ovarian cancer cells **(A)** Heatmap showing essentiality scores for four centriolar replication factors from 42 ovarian cancer cell line shRNA screens. Blue represents high essentiality (growth suppression) while red represents lower essentiality. STIL was essential in a higher proportion of cell lines than PLK4, while SASS6 and CENPJ were not found to be essential in the screens. **(B)** STIL mRNA levels (RNAseq) correlate with the histologic grade of ovarian cancer in the TCGA dataset, as determined using the cBioPortal (http://www.cbioportal.org).

To identify potential new therapeutic targets in ovarian cancer, we analyzed whole-genome lentivirus-based shRNA dropout screens [28]. As mitosis is a proven target for ovarian cancer therapy [14, 15, 17, 20, 29], we were interested in identification of centrosomal genes essential for ovarian cancer cell growth. We observed that STIL was essential for growth in 10 of the 42 ovarian cancer cell lines tested. Furthermore, some inhibition of growth (blue at Figure 1A) was observed in most of the cell lines exposed to STIL shRNA. Ovarian cancer cells were more sensitive to the depletion of STIL than to other centriolar replication factors including PLK4, SASS6 and CENPJ (Figure 1A and Supplementary Table 1). Analysis of published TCGA data [30] revealed that STIL is ubiquitously expressed in ovarian cancer and that its mRNA levels significantly correlate with a more advanced histological grade (Figure 1B).

**Figure 2:**
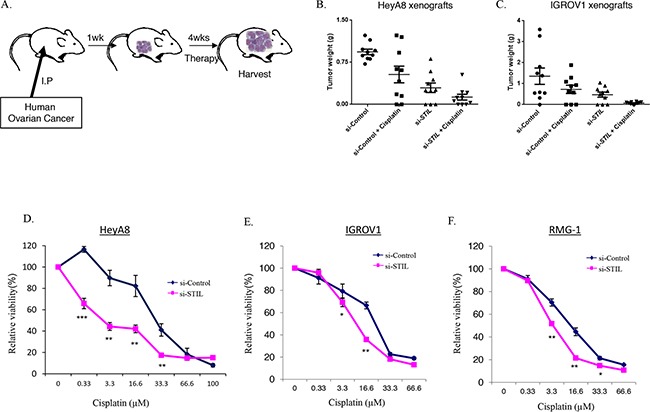
Therapeutic cooperation between STIL depletion and cisplatin in ovarian cancer **(A)** Illustration of the orthotopic models used for the *in-vivo* experiment: HeyA8 or IGROV1 cells were injected intraperitoneally (IP) to female nude mice. 8 days later, mice were divided into 4 groups and treatment was started by injecting siRNA/DOPC-nanoparticles (150μg/kg) twice a week and cisplatin (80μg/mouse) once a week intraperitoneally. Mice were treated for 4-5 weeks, sacrificed and tumors were excised and weighed. **(B)** Mean tumor weight in HeyA8 model, and **(C)** IGROV1 model. Values are means ± standard error. Main effect in 2-way ANOVA: p=0.004 for HeyA8 cells and p=0.028 in IGROV1 cells. **(D)** HeyA8 **(E)** IGROV1 **(F)** or RMG-1 cells were transfected with specific siRNA duplexes targeting the STIL gene (si*STIL*) or with a non-specific siRNA control (siControl). 48h post transfection the cells were treated with different concentration of cisplatin for additional 48h. Then, cells viability was measured by MTT assay. Data show the means of two independent experiments (*, p<0.05; **, p<0.01; ***, p<0.001 Student two-tailed unpaired t-test).

To validate the shRNA screen, we first confirmed the activity of STIL siRNA on ovarian cancer cell lines (for sequences see materials and methods and for confirmation of knockdown and activity see Supplementary Figure 1). We next evaluated the therapeutic efficacy of *STIL* siRNA *in-vivo* using well-characterized orthotopic ovarian cancer mouse models (Figure 2A) [15, 31, 32]. To simulate the treatment of advanced small-volume disease, therapy was initiated one week after tumor cell injection. We silenced *STIL* using intraperitoneal injection of *STIL*-specific siRNAs incorporated into DOPC nanoliposomes. Treatment with si*STIL-*DOPC alone resulted in 69% (HeyA8) and 65% (IGROV1) reduction in tumor burden compared to siControl-DOPC treated mice (Figure 2B, C). Animals treated with cisplatin alone (standard drug for treatment of ovarian cancer) showed 43% (HeyA8) and 47% (IGROV1) tumor reduction compared to siControl-DOPC treated groups. Combination therapy of si*STIL-*DOPC with cisplatin showed a significant reduction of tumor burden (HeyA8- 83%; IGROV1- 95%) compared to siControl-DOPC treated mice (Figure 2B, C, p=0.004 for HeyA8 and p=0.028 for IGROV1 in 2-way ANOVA). There was no obvious toxicity noted in the animals during the course of treatment, as assessed by changes in behavior, feeding habits, mobility and mean body weight (Supplementary Figure 2). Thus, siRNA suppression of STIL is effective for ovarian cancer therapy *in-vivo*.

### STIL depletion enhances sensitivity to DNA damaging agents

The therapeutic cooperation between STIL siRNA and cisplatin observed in the preclinical cancer models (Figure 2) raised the possibility that STIL depletion enhances the sensitivity of ovarian cancer cells to DNA damaging agents. Indeed, knockdown of STIL significantly enhanced the sensitivity to cisplatin in HeyA8, IGROV1 and RMG1 ovarian cancer cells (Figure 2D–2F) reducing the IC50 from a mean of 19μM (range: 13.3-30μM) to 6μM (range: 3.3-10μM; P<0.01 two tailed T-test). In contrast, there was no treatment sensitization with the microtubule poison paclitaxel (Supplementary Figure 3). Thus, STIL knockdown sensitizes ovarian cancer cells to DNA damaging chemotherapy.

**Figure 3:**
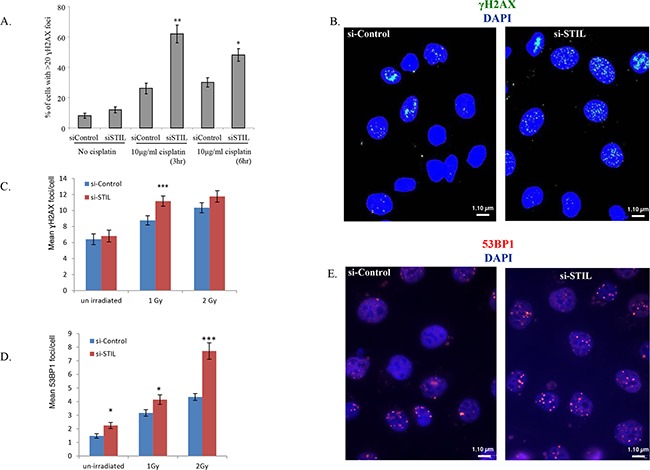
STIL depletion enhances γH2AX and 53BP1 foci in response to DNA damage **(A)** IGROV1 cells were transfected with siRNA targeting STIL or control, and 48 hrs later were treated by 10μg/ml cisplatin for 3 or 6 hr, after which cells were fixed and stained for γH2AX. Bar plots show the mean of two independent experiments, ± SEM, (*p<0.05; **p<0.01 in Student's two tailed unpaired t-test). **(B)** Representative images of cells 3h after cisplatin treatment. Magnification x60. **(C)** Same as in (a), except IGROV1 cells were treated with increasing doses of ionizing irradiation instead of cisplatin. γH2AX foci **(C)** or 53BP1 foci **(D)** were counted 24h after irradiation in 200 cells from each treatment. The mean number of foci per cell is shown. *P<0.01, **P<0.05, ***P<0.001 in Student's 2-sided unpaired T-test. **(E)** Representative images of 53BP1 foci in cells 4hrs after IR. Magnification x60.

Given the above findings, we next examined whether STIL depletion enhances the DNA damage conferred by DNA damaging agents. Ovarian cancer cells were either transfected with siControl or with si*STIL* and treated with 10μg/ml (33.3μM) cisplatin 48h later. Cells were stained for γH2AX 3 and 6 hours after initiation of treatment with cisplatin. There was a significant increase in γH2AX foci in STIL depleted cells treated with cisplatin (Figure 3A–3B). As platinum compounds cause DNA double-strand breaks (DSB) only indirectly, we further examined the effect of STIL depletion on DNA damage caused by ionizing (X-ray) radiation (IR). γH2AX foci increased after treatment with IR of two different ovarian cancer cell lines in a dose dependent manner (Figure 3C and Supplementary Figure 4). Similarly, we observed increased nuclear foci of p53-binding protein 1 (53BP1) in cells treated by STIL siRNA and IR (Figure 3D-E). Interestingly, there was a slight increase in γH2AX and 53BP1 foci after STIL siRNA alone (Figure 3A and 3D), suggesting that STIL may also be required for maintaining genome integrity. Together, these results suggest that STIL silencing enhances persistent DNA breaks-associated nuclear foci after exposure to DNA damaging agents.

**Figure 4:**
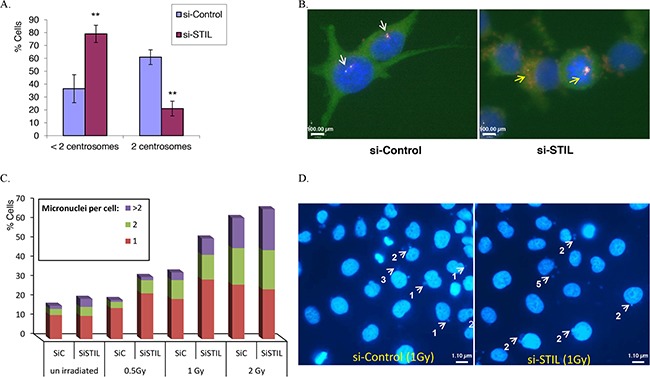
STIL silencing results in centrosomal aberrations and increased micronuclei following irradiation **(A, B)** HeyA8 cells were seeded on coverslips, transfected with siRNAs and stained for γ-tubulin (green) and pericentrin (red) 48h post siRNA transfection to quantify centrosome number. Nuclei were stained with DAPI. Most si-Control treated cells have 2 centrosomes per cell and undergo bipolar mitosis (white arrow), while most cells silenced for STIL have 0-1 centrosome per cell and some of the mitoses are monopolar (yellow arrow). Magnification x100. Bar plots show the mean of three independent experiments, ± STDEV (**p<0.005 in Student's two-tailed unpaired t-test.) **(C)** 48h post siRNA transfection, IGROV1 cells were irradiated (0.5, 1 and 2Gy), incubated for an additional 24h and then fixed and stained with DAPI. Micronuclei were counted in about 300 cells from each treatment, and the percentages of cells with1, 2 or more than 2 micronuclei were calculated. Shown is an average of two independent experiments. Two-tailed unpaired T-tests performed for the percentage of cells with more than 1 micronuclei: un-irradiated p=0.51, 0.5Gy p=0.06, 1Gy p= 0.025, 2Gy p= 0.44. **(D)** Representative images of micronuclei (arrows) from si-STIL or si-control cells 24h after 1Gy IR. Magnification x60.

We and others have previously reported that STIL is essential for centrosomal replication and that its depletion causes centrosomal abnormalities manifested by decreased centrosomal number and abnormal mitoses [1, 2, 4] (Figure 4A, B and Supplementary Figure 5). Centrosomal aberrations caused by abnormalities in centrosomal genes result in small chromosomal segregation errors, leading to chromosomal aneuploidy [23, 33]. We therefore examined the effect of STIL depletion on chromosomal aneuploidy in ovarian cancer cells two days after transfection with siRNA against STIL (Table 1). Interphase fluorescent *in situ* hybridization (FISH) was independently performed for 12 chromosomes and the chromosomal numbers were determined in 400 cells per each probe (a total of 4800 cells counted). Large deviations (≤2 or ≥6) in the modal number for specific chromosomes were detected for 8 of 12 chromosomes in STIL knocked-down cells (Table 1 and Supplementary Table 2, p < 0.05, Fisher's exact test). Thus, depletion of STIL causes chromosomal aneuploidy.

**Table 1:** STIL silencing in IGROV1 cells results in chromosomal aneuploidy

Chromosome	Less than 4 signals(hypoploidy)		4 signals(normal karyotype)		More than 4 signals(hyperploidy)		Fisher exact test
	Si-Ctrl	Si-STIL	Si-Ctrl	Si-STIL	Si-Ctrl	Si-STIL	
#2	0	16	394	368	6	16	p<0.001
#4	4	10	394	375	2	15	p=0.001
#7	11	12	385	384	4	4	p=1.0
#8	2	12	392	372	6	16	p=0.002
#9	0	16	394	384	6	0	p<0.001
#10	37	39	361	348	2	13	p=0.013
#12	6	6	390	390	4	4	p=1.0
#15	0	20	394	378	6	2	p<0.001
#17	6	52	392	333	2	15	p<0.001
#18	37	5	361	382	2	13	p<0.001

Chromosome	Less than 3 signals(hypoploidy)		3 signals(normal karyotype)		More than 3 signals(hyperploidy)		Fisher exact test
	Si-Ctrl	Si-STIL	Si-Ctrl	Si-STIL	Si-Ctrl	Si-STIL	
#6	0	2	398	382	2	16	p<0.001
#X	0	0	374	380	26	20	p=0.448

IGROV1 cells were transfected with specific siRNA duplexes targeting the STIL gene (siSTIL) or with a non-specific siRNA as control (siCtrl). 48hrs post transfection the cells were collected and fixed, and FISH analysis for 12 chromosomes was performed according to a standard protocol [54]. 400 cells were counted for each chromosomal probe.

Pellman et al reported that chromosomal mis-segregation in mitosis causes DNA breaks and acquisition of DNA damage via the formation of micronuclei [34, 35]. Consistent with these reports, an increased number of micronuclei was observed in IGROV1 cells treated with si*STIL* and low dose IR (0.5-1Gy; Figure 4C-D, p< 0.05 for 1Gy, two tailed T-test). Thus the enhanced DNA damage caused by STIL depletion may be explained by centrosomal abnormalities, chromosomal aneuploidy and enhanced micronuclei formation in response to IR.

### STIL is not essential for major DNA repair pathways

**Figure 5:**
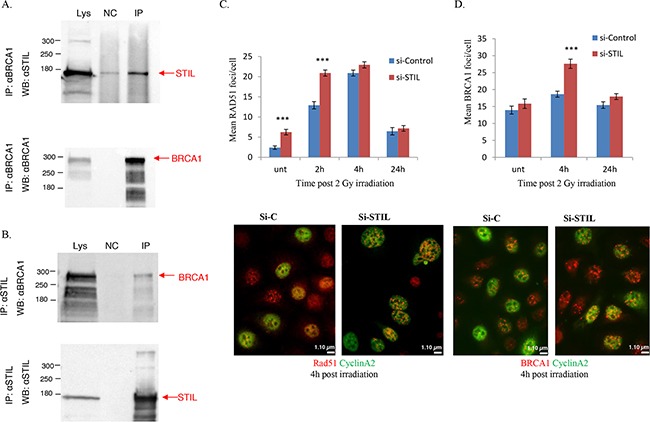
STIL and BRCA1 **(A, B)** STIL interacts with BRCA1: Flag-STIL and BRCA1 were transiently co-expressed in 293T cells. 48h later, cells were harvested, lysed and the indicated protein was precipitated with an antibody (anti BRCA1 in **A**, anti STIL in **B**) followed by protein A/G agarose beads. BRCA1 and STIL were detected by western blot. Lys- whole cell lysate, NC- bead-only controls, without antibody, IP- precipitation with the indicated antibody. Shown is a representative of three independent experiments. **(C, D)** STIL silencing increases BRCA1 and RAD51 foci following irradiation. 48h post siRNA transfection, IGROV1 cells were treated with 2Gy IR and RAD51 foci **(C)** or BRCA1 foci **(D)** in cyclin A2-positive cells were counted at the indicated time-points in 50-90 cells from each treatment. The mean foci/cell is presented. (***p<0.0005 in a two-tailed unpaired T-test). Shown is a representative of two independent experiments performed. Bottom: representative foci images. Magnification x60.

The observation that STIL KD enhances 53BP1 and γH2AX foci 24h after induction of DNA DSB (Figure 3C, D) could be explained by either ongoing DNA damage (e.g. due to ongoing chromosomal missegregation) and/or defects in DNA repair. BRCA1, a protein important in homologous recombination DNA repair (HRR), was reported to be localized to centrosomes [36, 37]. Ovarian cancers in patients with germline mutations in the BRCA1 or BRCA2 genes are more sensitive to DNA damaging agents such as cisplatin [38, 39]. We thus hypothesized that STIL depletion and the consequent centrosomal defects phenocopy BRCA1 depletion. This “BRCAness” could have explained the increased sensitivity to DNA damage in the absence of STIL. To test this hypothesis, we confirmed the binding of BRCA1 to STIL by co-immunoprecipitation in HEK293T cells (Figure 5A, B). To determine which domain of STIL binds to BRCA1 we repeated the co-immunoprecipitations of HEK 293T cells co-transfected with BRCA1 and various STIL mutants [1, 2]. All these mutants interacted with BRCA1. This suggests that STIL and BRCA1 binding is probably mediated by STIL N terminal domain either directly or as part of a larger protein complex (Supplementary Figure 7).

Next, we asked if STIL depletion was sufficient to cause a state of “BRCAness”. HRR is mediated by BRCA and RAD51 proteins [40]. Upon DNA damage, BRCA1, BRCA2 and RAD51 bind to DNA breaks. *Decreased* reduction in RAD51 foci is characteristic of BRCAness [40]. We therefore examined the effect of STIL depletion on the number of RAD51 and BRCA1 foci after induction of DSB by IR. Surprisingly, we observed an *increase* in both RAD51 and BRCA1 foci in the STIL depleted cells (Figure 5C, D). These observations suggest that STIL is not necessary for BRCA1/RAD51-dependent DNA repair. Interestingly, similar to the slight increases in γH2AX and 53BP1 foci after treatment with STIL siRNA alone (Figure 3A and 3D), RAD51 foci were also evident in STIL-depleted cells even in the absence of irradiation (Figure 5C).

**Figure 6:**
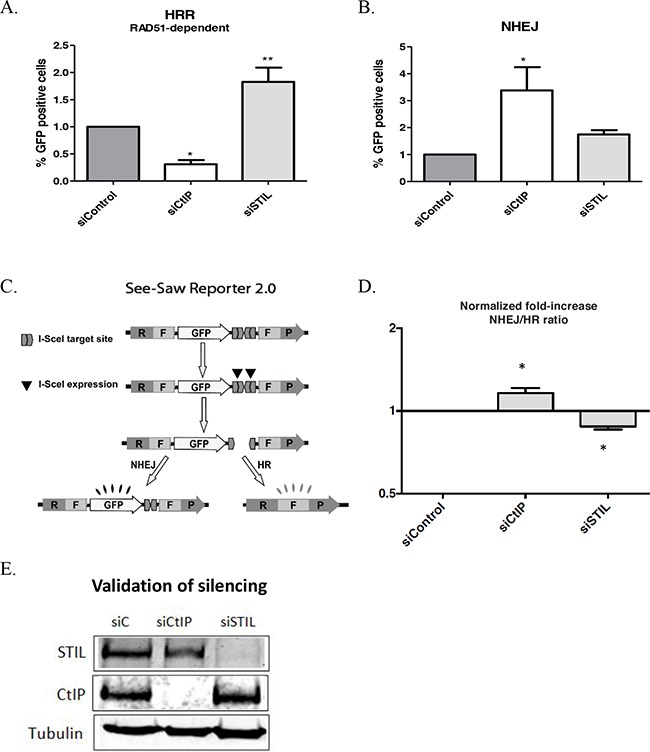
No major defects in repair of DNA double strand breaks (DSB) in STIL depleted cells **(A)** Effect of the indicated siRNA in the gene conversion assay which measures classical, RAD51-dependent homologous recombination. **(B)** Effect of the indicated siRNA in an assay which measures NHEJ. **(C)** Schematic representation of the SeeSaw 2.0 reporters [43]. A GFP open reading frame (ORF) is flanked by two truncated parts of an RFP ORF (RF and FP) sharing 302bp of homologous sequence. Two I-SceI-target sites in opposite orientation are present at the 3’ end of the GFP ORF. After generation of a DSB by ectopic I-SceI endonuclease, repair via Non Homologous End Joining (NHEJ) results in cells that retain GFP expression. Alternatively, repair via Homologous Recombination (HR) leads to a functional RFP ORF. **(D)** Ratio of NHEJ to HR repair in U2OS cells following siRNA transfection. Effects of siRNA against STIL, si-Control or against CtIP- a regulator of end resection required for homology-dependent DSB repair (positive control) are shown. The ratio of green to red cells in each condition was calculated. Increase NHEJ/HR ratio over the baseline value of 1.0 represents an imbalance between the two DSB repair pathways. An average of three experiments is shown (P=0.014 in Student's paired T-test). **(E)** Validation of STIL or CtIP silencing by western blot.

To further examine the effects of STIL on DNA repair, we determined the relative activity of the two main DNA repair pathways. U2OS cells bearing a single-copy integration of the reporters DR-GFP (HRR [41]), and EJ5 (NHEJ [42]) were used to analyze the different DSB repair pathways. As a positive control, we used siRNA against CtIP, which is a regulator of end-resection required for homology-dependent DSB repair. RAD51-dependent HRR was significantly increased in STIL-depleted cells (Figure 6A, E). These findings are consistent with the staining for BRCA1/RAD51 foci. NHEJ was not significantly changed in STIL-depleted cells (Figure 6B). A dual reporter assay [43] (Figure 6C) confirmed a small but statistically significant increase in HRR in STIL knock-down cells (Figure 6D, P=0.01).

We previously showed that STIL depletion slows the transition between G2 to mitosis [9]. Indeed, STIL silencing in HeyA8 and IGROV1 cells increased the G2/M fraction *in-vitro* and *in-vivo* (Supplementary Figure 6).

Together, these results indicate that STIL silencing disrupts both the cell cycle checkpoints and the balance between the DNA damage repair pathways.

## DISCUSSION

Despite significant activity of platinum and paclitaxel chemotherapy, advanced ovarian cancer is uniformly fatal [14]. Hence, novel therapies are urgently needed. Here, we report that STIL, a protein expressed in advanced ovarian cancer, may be such a novel therapeutic target.

STIL is one of the few mammalian proteins essential for *de-novo* centriolar formation and replication [1–4, 7, 44]. Centrosomes are often abnormal in cancer cells and centrosome amplification was shown to create chromosomal instability that promotes carcinogenesis and contributes to the aggressive behavior of ovarian cancer [18, 21]. Hence, centrosomal regulating proteins have been considered as targets for cancer therapy [22, 24, 25]. For example, we have previously shown that the centrosomal mitotic kinase SIK2 may be such a target [15].

Yet a recent study with a highly specific chemical inhibitor of PLK4, another critical centriolar replication factor, demonstrated that cancer cells can survive without centrioles [45]. Interestingly, in our shRNA screens (Figure 1A), PLK4 was essential for survival of a subset of ovarian cancer cell lines, suggesting a possible difference between enzymatic and non-enzymatic functions of PLK4. The importance of STIL as a scaffold for multiple proteins required for centrosome and cilia signaling, possibly explains why STIL was seen as the most essential in the shRNA screens of all centriolar replication factors (Figure 1A). STIL, however, is not essential for survival of non-neoplastic cells as demonstrated by the survival of STIL^−/−^ embryonic stem cells and fibroblasts [1, 12].

We also observed that STIL depletion enhanced the extent of DSB caused by DNA-damaging agents, and ultimately, cytotoxicity. There is conflicting data regarding the potential association between centrosomal defects and DNA damage. Loss of centrosomes in non-transformed cells was not associated with increased sensitivity to DNA damaging agents [46, 47]. In contrast, Pellman's lab has shown that in cancer cells, chromosomal missegregation caused by centrosomal abnormalities lead to the formation of micronuclei and to DNA damage. [34, 35, 48]. We show here that depletion of STIL in ovarian cancer cells caused chromosomal aneuploidy and the formation of micronuclei that might have caused the increased sensitivity to DNA damage.

The increased DNA damage in STIL-depleted cells exposed to DNA-damaging agents was unrelated to defects in DNA repair, or at least in the two major mechanisms of HRR and NHEJR. We have observed here that the proteins associated with HRR (BRCA1, Rad51) and NHEJ (53BP1) are retained in larger quantities upon STIL depletion. Consistent with these observations, HRR was increased in these cells. Interestingly, we demonstrate for the first time that STIL and BRCA1 are present in a protein complex. It is tempting to speculate that in the absence of STIL more BRCA1 is available for recruitment to DNA damage sites. Alternatively, the increased length of the G2 to M transition in STIL KD cells may increase the time available for DNA repair.

Several recent publications have highlighted the interplay between 53PB1, centrosomes loss and activation of p53 [49–51]. Specifically they demonstrated that 53BP1 mediates p53-induced cell cycle arrest following centrosomal loss, by stabilizing p53. Yet it is unlikely that this pathway explains the cytotoxicity of STIL depletion in ovarian cancers. P53 is mutated in most ovarian cancers (including IGROV1) and we did not find any correlation between the essentiality of STIL in the shRNA screen (Figure 1A) and the status of P53 in the ovarian cancer cell lines.

Our most significant finding is that combination of weekly systemic cisplatin with intraperitoneal delivery of *STIL* siRNA was a highly effective therapy in two preclinical models of advanced ovarian cancer. As the first clinical trial with siRNA packed in DOPC is ongoing (NCT01591356), targeting STIL with siRNA may be clinically feasible.

## MATERIALS AND METHODS

### Plasmids

We used Flag-hSTIL and Flag-hSTIL deletion mutants in a lentiviral vector previously described [1]. pcDNA3-hBRCA1 was a kind gift of Dr. Ido Wolf.

### Drugs

Cisplatin and paclitaxel were obtained from Teva Pharmaceutical Industries Ltd.

### Antibodies

The following antibodies were used for western blot: rabbit anti STIL A302-441A1 (Bethyl Laboratories), mouse anti BRCA1 (Santa Cruz, sc-6954) and for loading controls mouse anti vinculin (Millipore MAB3574) and mouse anti alpha-Tubulin (Sigma T9026). Secondary antibodies were peroxidase-conjugated goat anti rabbit and goat anti mouse (Jackson Immunoresearch, #111-035-144 and #115-035-146). The following antibodies were used for immunofluorescence: mouse anti gamma- tubulin (Abcam ab11316 and Sigma T5192), rabbit anti pericentrin (Abcam, ab4448), rabbit anti phospho Histone3 (Abcam, Ab32107), mouse anti yH2AX (Ser139, Merck-Millipore #05-636), rabbit anti 53BP1 (H-300, Santa Cruz, sc-22760), rabbit anti Rad51 (Calbiochem, #PC130), rabbit anti BRCA1 (Millipore, #07-434) and mouse anti cyclinA2 ([6E6], Abcam, ab16726). Secondary antibodies were donkey anti rabbit Alexa Fluor 568 and donkey anti mouse Alexa Fluor 488 (Invitrogen).

### Cell culture

The HeyA8 human ovarian cancer cell line, was obtained from Dr. Isaiah Fidler (M. D. Anderson Cancer Center, Houston, TX). The IGROV1 and RMG-1 cell lines were obtained from the institutional Cell Line Core laboratory (MD Anderson policy ACA#1044). The IGROV1-CP20 cell line was developed by sequential exposure of the IGROV1 cell line to increasing concentrations of cisplatin. Cell line authentication was performed by the Cell Line Core Facility at The University of Texas MD Anderson Cancer Center at least once per year.

IGROV1 (used for the *in vitro* experiments), IGROV1cp20 (used for the *in-vivo* experiments), HeyA8 and RMG-1 cells were cultured in RPMI 1640 supplemented with 10% FCS at 37°C and 5% CO_2_ atmosphere.

### RNA interference

#### *In-vitro* studies

For siRNA-mediated suppression of human STIL, the following oligonucleotide sequences were used: sequence “1207” 5’-GGGCTTGCTGTTTGCGATACATATT-3’ (Invitrogen) or sequence “STIL#1” 5’-GTTGTGAA CTGAGCGCTGA-3’ (Sigma). Mission siRNA Universal Negative Control #1 (Sigma) was used as a control. siRNAs were introduced into cells using SiImporter siRNA transfection reagent (Millipore, #64-101), according to the manufacturer's instructions. 48h after transfection, cells were harvested for WB or RT-PCR analysis of STIL levels. For *in-vivo* delivery, siRNA oligos were incorporated into neutral liposomes (DOPC) as previously described [32].

The shRNA screens were performed as described previously [28]. Briefly, each cell line was infected with the RNAi Consortium (TRC) genome-wide lentiviral shRNA library at an MOI of 0.3. Cells were puromycin-selected, representative ‘T0’ samples were collected, and the remaining cells were split into three flasks. Triplicate cultures were then grown out for three to six passages, at which point genomic DNA was collected, shRNA barcode sequences were amplified by PCR, and hybridized to a custom microarray for quantification. Hairpin dropout scores (shRNA Activity Ranking Profile, or 'SHARP’ scores) were calculated as previously described [28] and combined into a Gene Activity Ranking Profile(‘GARP’) score. More negative GARP scores indicate a higher degree of essentiality for the targeted gene. STIL was determined to be essential where the GARP P-value was less than 0.05.

### Immunostaining

For detection of yH2AX, 53BP1, RAD51, BRCA1 nuclear foci and micronuclei, cells were seeded on coverslips, transfected with siRNA against STIL or Control using SiImporter (described above), and 2 days later treated with a DNA-damaging agent (cisplatin or irradiation). Staining was performed as follows: Coverslips were washed in PBS, fixed in 4% Paraformaldehyde for 10 minutes and permeabilized in 2% PFA and 0.5% Triton for 10 minutes. After washes in PBS and blocking in 10% Normal Donkey Serum (NDS) in PBST (PBS+0.5% Tween) for 45 minutes, they were incubated with primary antibodies (anti yH2AX 1:100, anti 53BP1 1:200, anti Rad51 1:500, anti BRCA1 1:2000 and anti CyclinA2 1:100) for 2h in 10%NDS, washed in PBST and then incubated with secondary antibodies (donkey anti rabbit Alexa Fluor 568 and donkey anti mouse Alexa Fluor 488, both 1:1000) for 45 minutes in the dark. After washing with PBST they were mounted using ProLong Gold antifade reagent with DAPI (Molecular probes, #P36935). After 3 days, images of 50-300 cells from each treatment were taken and foci were counted using a Nikon fluorescence microscope.

For detection of the centrosomal markers gamma tubulin and pericentrin, staining was performed 48h after siRNA treatment transfection as described above, except that fixation was done in methanol:acetone (1:1) at -20°C without the additional permeabilization step. Images were acquired using an Olympus IX81 fluorescence microscope, 100 cells were counted and analyzed for each experiment.

### Co-immunoprecipitation

HEK293FT cells were co-transfected with Flag-hSTIL and hBRCA1-encoding plasmids. 48h later, cells were lysed in immunoprecipitation buffer (10mM Tris pH 7.5, 150mM NaCl, 0.5% Triton-X100) with fresh protease inhibitors (Complete mini, Roche), incubated 15 minutes on ice and centrifuged for 15 minutes at 12,000g. The supernatant was split into two tubes: “IP” containing the precipitating antibody (either anti STIL or anti BRCA1) and “NC” containing no antibody. The lysates were rotated gently overnight at 4°C. Then, 30μl protein A/G agarose beads (Santa Cruz) were added for an additional 1.5h at 4°C to both the IP and negative control tubes. Samples were washed 4 times with NET- 2 washing buffer (50 mM Tris-HCl, 150 mM NaCl), re-suspended in 40μl of sample buffer x1, denatured at 95°C for 5 minutes and analyzed by western blot using anti-BRCA1 and anti-STIL antibodies.

### Chromosomal aneuploidy analysis

IGROV cells were transfected with specific siRNA duplexes targeting the STIL gene (*siSTIL*) or with a non-specific siRNA as control (siControl). 48h post transfection the cells were collected, fixed, and interphase Fluorescence *in situ* hybridization(FISH) analysis using Satellite probes of Vysis, (Vysis Downers, Grove, IL) for 12 chromosomes was performed according to the standard protocol detailed in [52]. Slides were analyzed using an Olympus BH2 fluorescence light microscope equipped with a PlanApo 100x/1.4 oil-immersion objective, an appropriate spectral filter (BH2-TFC1 Triple Band filter DAPI/FITC/TRITC), and a 100W mercury arc lamp. 400 cells were counted for each probe.

### *In-vitro* cytotoxicity experiments

For the combined experiments using siRNA and drug (cisplatin or paclitaxel), cells were seeded in 24 wells at a confluence of 30% and transfected the next day as described in “RNA interference”. After 48h, drug was added to each well in duplicates, and 48h later viability was measured by counting viable cells under the microscope using trypan-blue, or using the MTT-based *in- vitro* toxicity assay(Sigma, TOX1), according to the manufacturer's protocol.

For the combined experiments using siRNA and ionizing radiation, the same protocol was used except cells were irradiated in increasing doses in an X-ray irradiator (Kimtron Polaris® 320).

### *In-vivo* studies

The female athymic nude mice for orthotopic ovarian cancer models were maintained as described earlier [31]. All mice were used in these experiments when they were 8 to 12 week old.

Long-term therapy experiments were performed using HeyA8 and IGROV1cp20 ovarian tumor models. Prior to injection, tumor cells were washed twice with PBS, detached by 0.1% cold EDTA, centrifuged for 5 minutes and reconstituted in Hanks balanced salt solution (Invitrogen, Carlsbad, CA). Cell viability was confirmed by trypan blue exclusion. Tumors were established by intraperitoneal (i.p) injection of 250,000 (HeyA8) and 1.0 x 10^6^ (IGROV1cp20) cells.

To assess the effects of siRNA therapy on tumor growth, treatment was initiated one week after i.p. injection of tumor cells. Two different *STIL* siRNA sequences were used: STIL#1 for HeyA8 and STIL #1207 for IGROV1cp20 as mentioned above. Mice were divided into 4 groups (n = 10 mice per group): (a) siControl-DOPC, (b) siControl-DOPC + cisplatin, (c) si*STIL-*DOPC, and (d) si*STIL*-DOPC + cisplatin. Mice were treated intraperitoneally with 150 μg/kgs iRNAs twice a week and cisplatin (80 μg/mouse) once weekly. Treatment was continued until mice in any group became moribund (typically 4-5 weeks following tumor cell injection). At the time of sacrifice, mouse weight, tumor weight, number of nodules, and distribution of tumors were recorded. Tumor tissue was harvested and either snap-frozen in liquid nitrogen for lysate preparation or fixed in formalin for paraffin embedding, or frozen in optimum cutting temperature medium (OCT; Miles, Inc., Elkhart, IN) to prepare frozen slides. The individuals who performed the necropsies, tumor collections, and tissue processing were blinded to the treatment group assignments.

To detect phospho Histone 3 (pH3) from frozen tumor tissues which were harvested 3days following siRNA treatment, frozen tumors were cut to 5 mm sections. Slides were dried for 30 minutes at room temperature, rehydrated in PBS for 5 minutes, fixed in ethanol at -20c for 10 minutes, incubated with blocking solution (5% normal goat serum and 0.5% Triton X-100 in PBS) for 1 h, and then incubated with rabbit anti-pH3 primary antibody for 4 h at room temperature. Slides were washed 3 times with PBS for 10 minutes and incubated with the secondary antibodies (Alexa Flour 568) for 2 h at room temperature, followed by mounting using ProLong Gold antifade reagent with DAPI (Invitrogen). 3 tumors from each group were analysed, images of 5 fields from each tumor were photographed using an Olympus IX81 fluorescence microscope.

### Gene conversion, non homologous end joining (NHEJ) and recombination */* NHEJ balance analysis

The analysis was performed as described previously [43]. Briefly, U2OS cells bearing a single copy integration of the reporters DR-GFP (Gene conversion;Homologous Recombination Repair, HRR [41]), EJ5 (NHEJ [42]) or SSR (NHEJ*/*recombination balance;[43]) were used to analyze the different DSB repair pathways. For each experiment, 60000 cells were plated in 6-well plates. One day after seeding, siRNA (si-control, si-CtIP or si-*STIL*) transfection was performed using Lipofectamine (according to the manufacturer's instructions). The next day, cells were infected with a lentivirus encoding the I-SceI endonuclease and labeled with Blue Fluorescent Protein (BFP) using a multiplicity of infection (M.O.I) of 10. 24h later, the media was changed. The next day, cells were fixed using 4% paraformaldehyde and analyzed in a BD FACS Aria cell sorter. For the HRR*/*NHEJ balance, the ratio between green and red cells in each condition was calculated. Data represent a minimum of three sets of experiments performed in duplicates.

### Cell cycle DNA content analysis

48h following siRNA transfection, HeyA8 and IGROV1 cells were collected, fixed in ice-cold EtOH over-night and stained the following day with Propidium Iodide (PI, Sigma P4864). Analysis was performed on the Gallios flow cytometer and data were elaborated using Kaluza software (Beckman Coulter).

### Statistics

Data obtained from multiple experiments were reported as the mean ± SEM. Significance levels were determined by Student *t* test, analysis of variance (ANOVA) analysis or Fisher's exact test (specifically indicated in each experiment).

## SUPPLEMENTARY MATERIALS FIGURES AND TABLES


