# Supplementary material for: Targeting the centriolar replication factor STIL synergizes with DNA damaging agents for treatment of ovarian cancer
Source: Oncotarget. 2017 Mar 10;8(16):27380–92. doi: 10.18632/oncotarget.16068 (PMC5432342; doi:10.18632/oncotarget.16068)
Supplement: Supplementary file 1 [file oncotarget-08-27380-s001.pdf]

Targeting the centriolar replication factor STIL synergizes with DNA damaging agents for treatment of ovarian cancer

SUPPLEMENTARY MATERIALS

SUPPLEMENTARY FIGURES AND TABLES

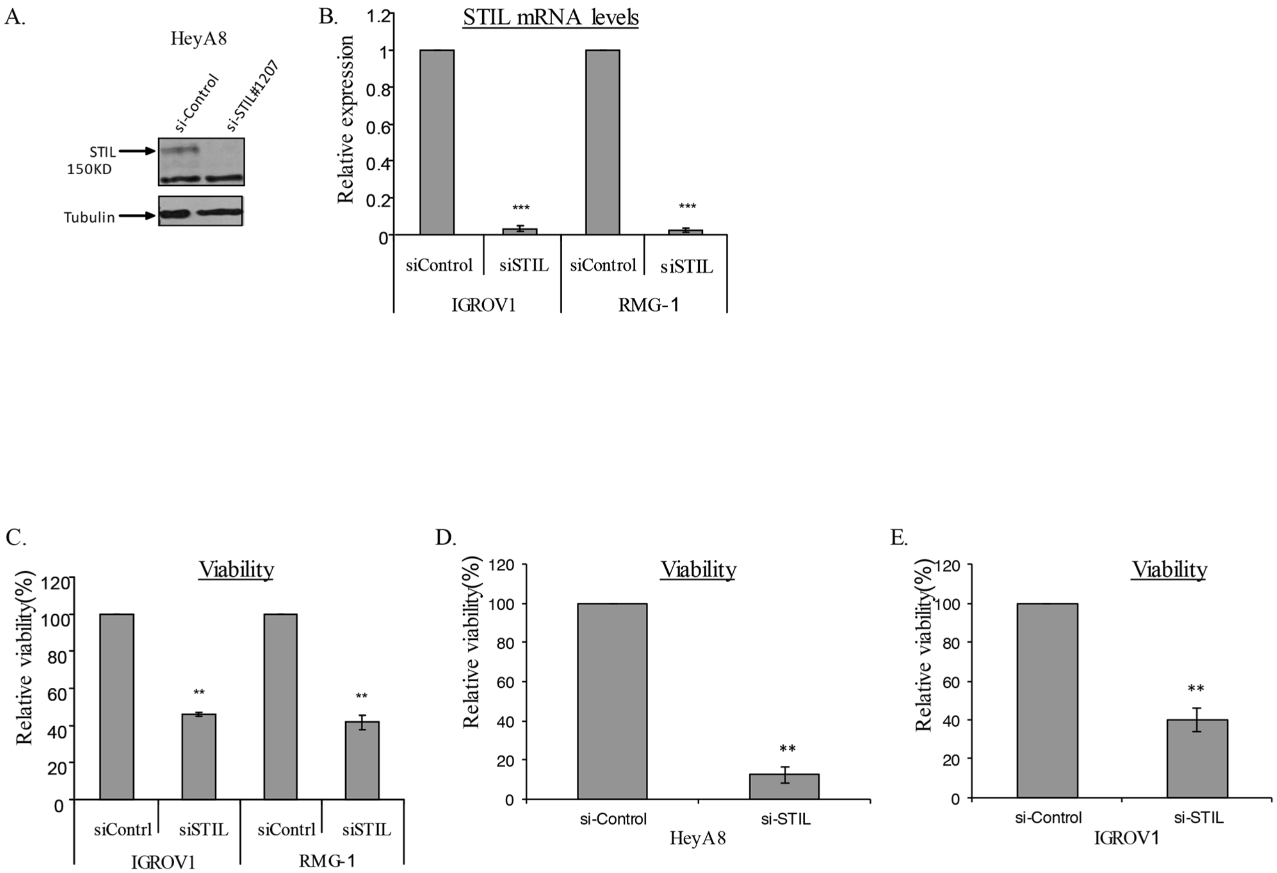

Supplementary Figure 1: siRNA against STIL reduces the survival of ovarian cancer cell lines. HeyA8, IGROV1 and RMG-1 cell lines were transfected with specific siRNA duplexes targeting the STIL gene (siSTIL) or with a non-specific siRNA as control (siControl). 48h post transfection, a 2nd transfection was performed, followed by MTT assay 3 days later. **(A)** Validating STIL silencing in HeyA8 cells (western blot) and in IGROV1 and RMG-1 cells **(B)** by Q-RT-PCR. **(C)** Relative viability as determined by MTT. **(D,E)** The same experiment as C was performed for **(D)** HeyA8 and **(E)** IGROV1, only the measurement was done by viable cell count and not MTT. Results are representative of three independent experiments (**P<0.01, two-tailed unpaired T-test).

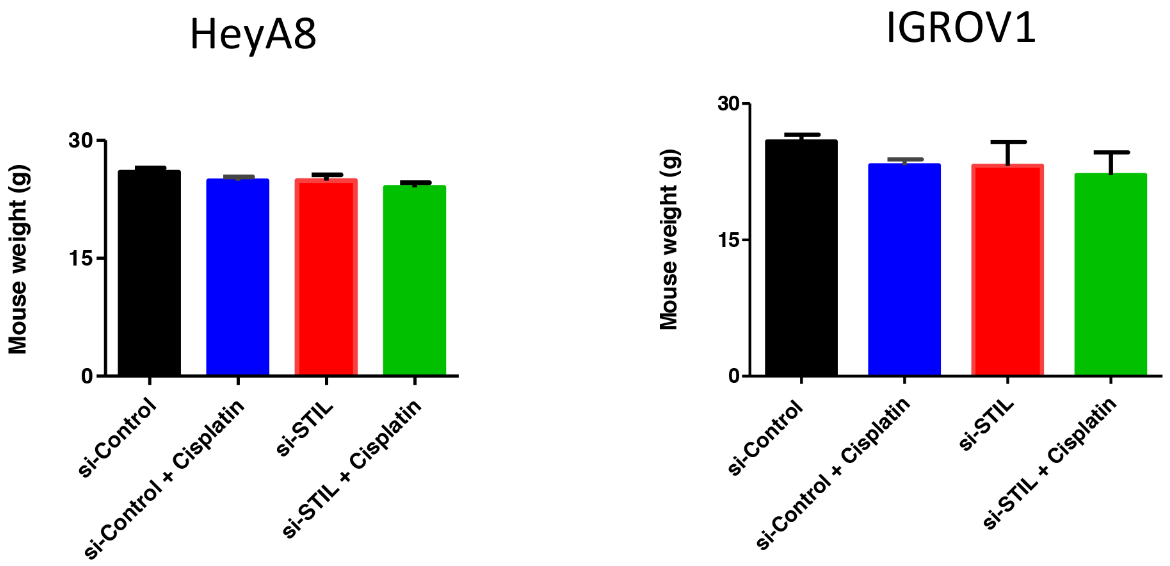

Supplementary Figure 2: Weight of xenografted mice was unaffected by *in-vivo* si-RNA and cisplatin treatments. HeyA8 or IGROV1 cells were injected intraperitoneally (IP) into female nude mice. 8 days later, mice werre divided into 4 groups and treatment was started by injecting siRNA/DOPC-nanoparticles (150µg/kg) twice a week, and cisplatin (80µg/mouse) once a week intraperitonally. Mice were treated for 4-5 weeks, sacrificed when become moribund in any group and tumors were excised and weighed. Mean mice weights for HeyA8 (left) and IGROV1 (right) xenografts.

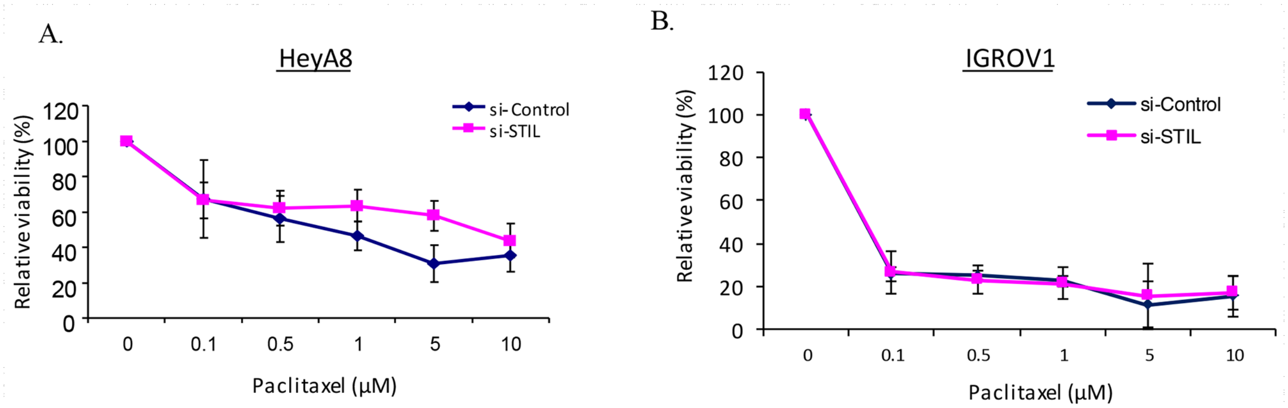

Supplementary Figure 3: STIL depletion does not sensitize ovarian cancer cells to paclitaxel. HeyA8 **(A)** and IGROV1 **(B)** cell lines were transfected with specific siRNA duplexes targeting the STIL gene (si*STIL*) or with a non-specific siRNA control (siControl). 48h post transfection the cells were treated with different concentration of paclitaxel for additional 48h. Then, cells viability was measured by MTT assay. Data are an average of three independent experiments.

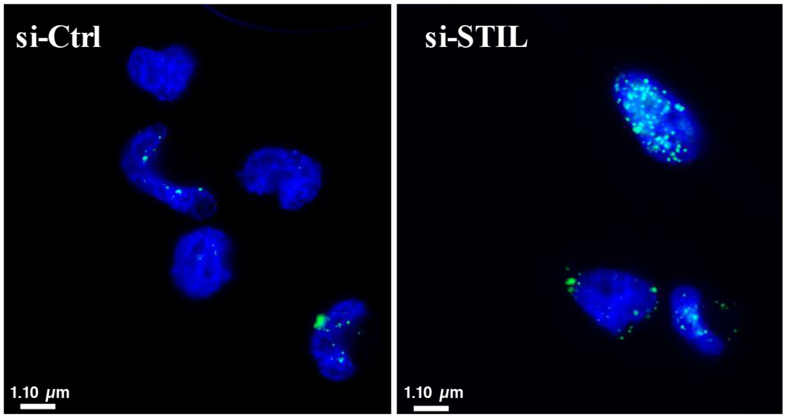

Supplementary Figure 4: γH2AX foci in HeyA8 cells 3h following 2Gy irradiation. STIL-depleted HeyA8 cells were irradiated (2Gy) and stained for γH2AX 3h later. An increase in γH2AX foci in STIL silenced cells versus control siRNA treated cells was observed. Magnification x100.

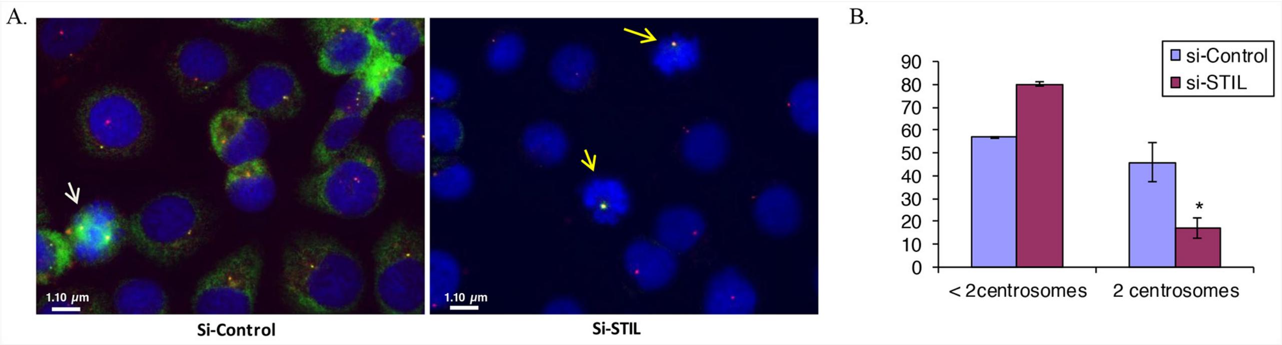

Supplementary Figure 5: siRNA against STIL causes centrosomal and mitotic aberrations in IGROV1 cells. IGROV1 cells were seeded on coverslips, transfected with siRNAs and stained for gamma tubulin (green) and pericentrin (red). Nuclei were stained with DAPI (blue). **(A)** Most siControl-treated cells have two centrosomes per cell and undergo bipolar mitosis (white arrow), while most cells silenced for STIL have one centrosome per cell and undergo monopolar mitosis (yellow arrow). Magnification x100. **(B)** Bar plots show the mean of two independent experiments, ± STDEV, (*$p<0.05$ in Student's two-tailed unpaired t-test).

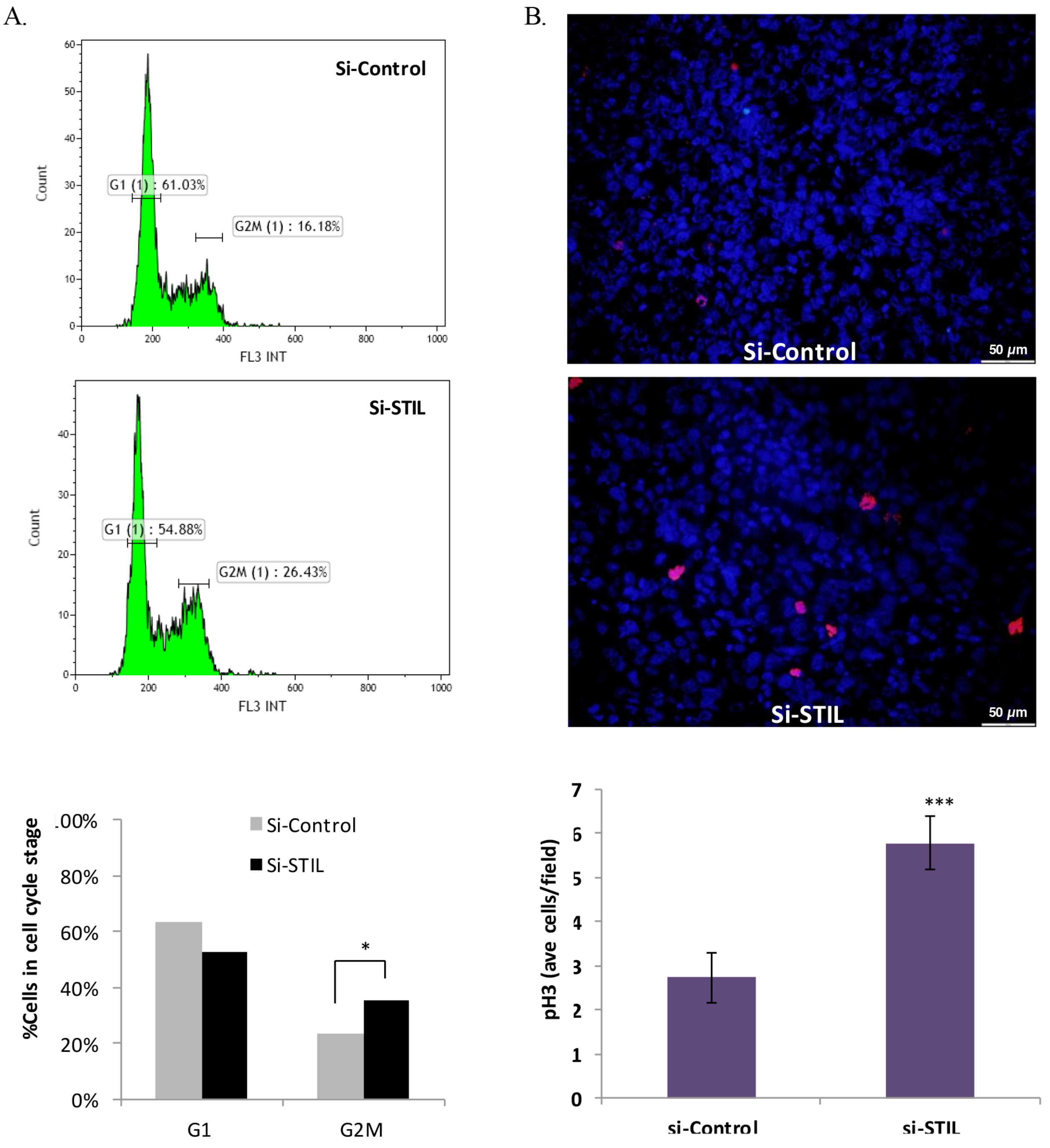

Supplementary Figure 6: STIL silencing alters the cell cycle by slowing the G2/M transition *in-vitro* and *in-vivo*. (A) Cell cycle analysis of IGROV1 cells stained with PI shows an elevated G2/M fraction following STIL silencing. Shown are histograms from a representative experiment (top), and a bar plot representing an ave. of 3 independent experients (bottom), *P=0.036 in a student's 2 tailed paired t-test. **(B)** HeyA8 ovarian cancer xenografts treated with 2 injections of the indicated siRNA/DOPC-nanoparticles were harvested 1 day after the last injection, snap frozen, sectioned and stained for phospho histone 3 (pH3, red) and DAPI (blue). 3 tumors from each group were analysed, 5 fields of each tumor were photographed and the number of positive pH3 cells counted. Shown is a representative image of each group (top) and mean pH3 cells per field (bottom). ***P<0.005 in a student's 2 tailed un-paired t-test.

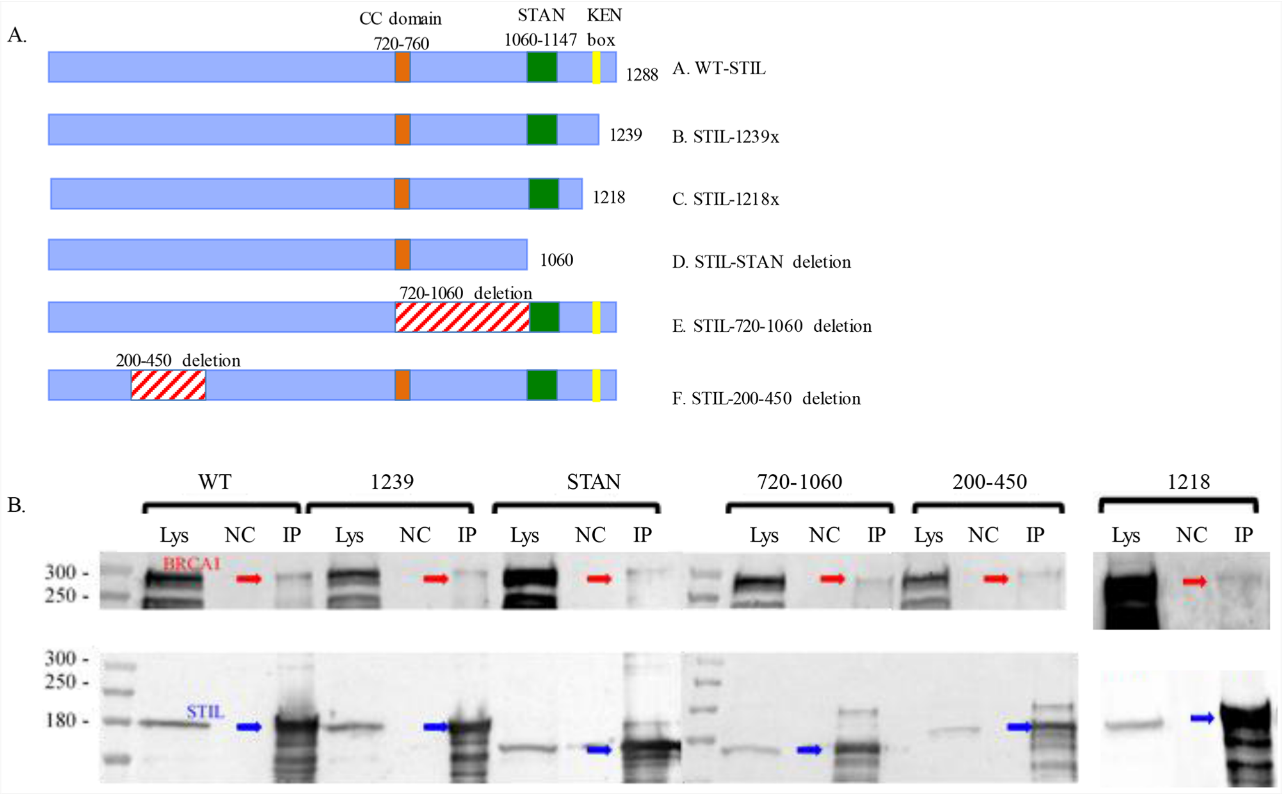

Supplementary Figure 7: The interaction between STIL and BRCA1 is not mediated by the C-terminal of STIL. Flag-wt-STIL or the indicated deletion mutants shown in **(A)** and BRCA1 were transiently co-expressed in 293T cells. **(B)** 48h later, cells were harvested, lysed and STIL (blue arrows) was precipitated with an antibody followed by protein A/G agarose beads. BRCA1 (red arrows) was detected by western blot. Lys- whole cell lysate, NC- bead-only controls, without antibody, IP- precipitation with the indicated antibody. Shown is a representative experiment of 2 performed.

Supplementary Table 1: List of Ovarian cancer cell lines included in the shRNA screen and the essentiality score.

See Supplementary File 1

Supplementary Table 2: FISH analysis of 12 chromosomes from IGROV1 cells silenced for STIL (Si -STIL) or si-Control (Si-Ctrl).

See Supplementary File 2
